# Increased decision thresholds trigger extended information gathering across the compulsivity spectrum

**DOI:** 10.1038/s41398-017-0040-3

**Published:** 2017-12-18

**Authors:** Tobias U. Hauser, Michael Moutoussis, NSPN Consortium, Peter Dayan, Raymond J. Dolan

**Affiliations:** 1 Wellcome Trust Centre for Neuroimaging, University College London, London, WC1N 3BG United Kingdom; 2 Max Planck UCL Centre for Computational Psychiatry and Ageing Research, London, WC1B 5EH United Kingdom; 3 Gatsby Computational Neuroscience Unit, University College London, London, United Kingdom

## Abstract

Indecisiveness and doubt are cognitive phenotypes of compulsive disorders, including obsessive–compulsive disorder. Little is known regarding the cognitive mechanisms that drive these behaviours across a compulsivity spectrum. Here, we used a sequential information gathering task to study indecisiveness in subjects with high and low obsessive-compulsive scores. These subjects were selected from a large population-representative database, and matched for intellectual and psychiatric factors. We show that high compulsive subjects sampled more information and performed better when sampling was cost-free. When sampling was costly, both groups adapted flexibly to reduce their information gathering. Computational modelling revealed that increased information gathering behaviour could be explained by higher decision thresholds that, in turn, were driven by a delayed emergence of impatience or urgency. Our findings show that indecisiveness generalises to a compulsivity spectrum beyond frank clinical disorder, and this behaviour can be explained within a decision-theoretic framework as arising from an augmented decision threshold associated with an attenuated urgency signal.

## Introduction

A tradeoff between certainty and the time spent on option evaluation is a crucial, and non-trivial aspect of decision making^1–3^. Spending too little time on an important decision (e.g., whom to marry) can be highly deleterious, as can spending too much time making relatively unimportant decisions (e.g., where to buy lunch).

Patients with obsessive-compulsive disorder (OCD) appear to suffer the latter affliction, and can be described as both indecisive and intolerant of uncertainty^4,5^. Indeed, many symptoms can be thought of in terms of extended information gathering behaviour that is the complement of OCD subjects’ need for certainty (e.g., checking that all windows are closed). Despite a rich symptomatic description in the literature there is a dearth of experimental studies that decompose this behaviour. Most studies support a link between OCD and increased information gathering behaviour^6–10^, although not unequivocally^11–13^. In a recent computational study, we showed that adolescent patients with OCD express increased information gathering due to an increased decision threshold and altered subjective sampling costs^7^.

A fundamental problem in many clinical studies is the observation that patients often suffer additional comorbidities, notably (sub-)clinical levels of depression and anxiety as well as potential influences from current or past medication. There is also concern in relation to the explanatory utility of categorical psychiatric diagnoses^14,15^ that has led to a re-conceptualisation in terms of psychiatric dimensions^16^. In this latter framework patients with OCD represent those at an extreme of a compulsivity spectrum while allowing for the presence of considerable compulsivity heterogeneity within an otherwise ‘healthy’ population.

We sought to examine whether excessive information gathering behaviour was a marker of such a compulsivity spectrum rather than simply the expression of a categorical disease state. We recruited forty young adults from a large population-representative sample from whom we had collected psychiatric and other health-related information. We followed a targeted recruitment approach, in which we selected non-clinical subjects scoring either high or low on an obsessive-compulsive symptom scale, but matching these groups on other (psychiatric) dimensions, such as mood, anxiety, age, gender, and intellectual abilities. This allowed us to overcome limitations in patient studies, such as those arising out of comorbidities or medication usage. Using a sequential information gathering task, we show that high compulsive subjects express similar behavioural and computational features as patients with OCD, consistent with the general idea of a compulsivity spectrum where pathology represents an extreme.

## Materials and methods

### Subjects

The goal of this study was to compare information gathering in subjects who differed in obsessive-compulsive symptoms (subsequently called ‘compulsivity’ for short, but this is not intended to imply an exclusion of obsessions), but were comparable on other psychiatric traits, in particular in relation to symptoms of depression and anxiety. This is important because a high comorbidity between OCD, depression and anxiety often renders a dissociation difficult for classic patient studies[17]. We thus used a large population-representative sample of young people in London and Cambridge (U-CHANGE study; *N* = 2409; www.nspn.org.uk ^18–20^), from whom we had collected questionnaire and health-related information. From this database, we recruited 20 adult subjects with low (21.40 ± 2.52 years) and 20 adults with high compulsivity scores (20.75 ± 2.34 years; group details see Table 1). Importantly, the low compulsive subjects were specifically selected so as to match the high compulsives on anxiety and depression scores, which allowed us to determine whether information gathering biases were specific to variation in the compulsivity spectrum.

**Table 1:** Subject characteristics

	Low compulsive group	High compulsive group	
Age*	21.40 ± 2.52	20.75 ± 2.34	t(38) = .85, *p* = .403
Gender (f/m)*	13/7	14/6	χ(1) = .114, *p* = .736
Handedness (r/l)	16/4	16/4	χ(1) = .0, *p* = 1.00
IQ (WASI total)	115.6 ± 10.9	115.4 ± 9.8	t(38) = .06, *p* = .952
PI-WSUR total*	5.3 ± 4.0	50.2 ± 18.3	t(38) = 10.74, *p* < .001
MFQ*	19.1 ± 8.9	19.4 ± 11.7	t(38) = .07, *p* = .942
RCMAS total*	20.7 ± 10.1	18.7 ± 10.7	t(38) = −.61, *p* = .545
BDI-II total	6.1 ± 4.2	8.7 ± 7.1	t(38) = 1.41, *p* = .166
STAI (trait)	38.3 ± 6.5	41.4 ± 11.3	t(38) = 1.05, *p* = .302
STAI (state)	33.5 ± 6.1	34.0 ± 9.4	t(38) = .20, *p* = .843
BIS	58.30 ± 6.87	59.04 ± 9.74	t(38) = −.28, *p* = .782
Intolerance of uncertainty	48.75 ± 15.14	58.80 ± 15.87	t(38) = −2.05, *p* = .047
FMPS total	98.02 ± 16.21	103.02 ± 17.35	t(38) = −0.94, *p* = .353

Subjects were recruited from a population-based database so that groups differed maximally on the compulsivity spectrum (PI-WSUR)^21^, but were matched for age, gender, depression (MFQ)^22^ and anxiety (RCMAS)^24^. The groups did not differ in depression (BDI-II)^27^, anxiety (STAI)^26^, impulsivity (BIS)^28^, handedness^30^ or intellectual abilities (WASI)^29^, as assessed on the day of the experiment. Groups differed in their intolerance of uncertainty^33^, but not in perfectionism (FMPS)^46^. (mean ± SD); *data used for recruiting participants

As an index of compulsivity, we used the total score of the revised Padua Inventory questionnaire (PI-WSUR)^21^. The PI-WSUR is an established questionnaire for assessing obsessive-compulsive traits with a high test-retest reliability and internal consistency^21^. The subjects with high compulsive scores were in the 91.62 ± 5.83 percentile of the U-CHANGE populations’ PI-WSUR distribution, whereas the low compulsive group scored on the 27.29 ± 16.55 percentile of this distribution.

To match groups for depressive mood, we recruited subjects based on the Mood and Feelings Questionnaire (MFQ)^22^, which has a high internal consistency and can be used as a screening for depression^23^. As a recruitment measure of anxiety, we used the Revised Children’s Manifest Anxiety Scale (RCMAS)^24^, again known to have good psychometric properties^25^. The recruited groups scored on both questionnaires well within the normal range of the population (MFQ: low compulsivity: 31.53 ± 13.64 percentile, high compulsivity: 30.13 ± 16.18; RCMAS: low compulsivity: 31.63 ± 12.06, high compulsivity: 28.71 ± 14.47), thus not being either particularly low or high in depressive and anxiety symptoms.

At the day of the assessment, we complemented these measurements with additional questionnaires of anxiety and depression that were specifically developed for >18 year olds, because both MFQ and RCMAS were primarily validated in children and adolescents. We thus collected the State and Trait Anxiety Inventory (STAI)^26^ and Beck Depression Inventory II (BDI-II)^27^. In addition, we collected further measures, such as impulsivity (Barratt Impulsiveness Scale, BIS)^28^, intelligence (matrix and vocabulary subtests of the Wechsler Abbreviated Scale of Intelligence, WASI)^29^, and handedness^30^. The groups differed in none of the measures (full details in Table 1), ensuring that a difference in compulsivity is the key discriminating feature between the groups.

Moreover, subjects were only recruited if they fulfilled the following additional inclusion criteria: no neurological or psychiatric diagnosis (self-reported screening question), over 18 years, living in London, absence of colour blindness. Data from these subjects has previously been reported, investigating a different task collected on the same occasion^20^. The study was approved by the UCL research ethics committee (No. 6218/001) and all subjects gave written informed consent. Subjects received monetary compensation for their participation, but this did not depend on the performance in the reported task.

### Task

**Figure 1:**
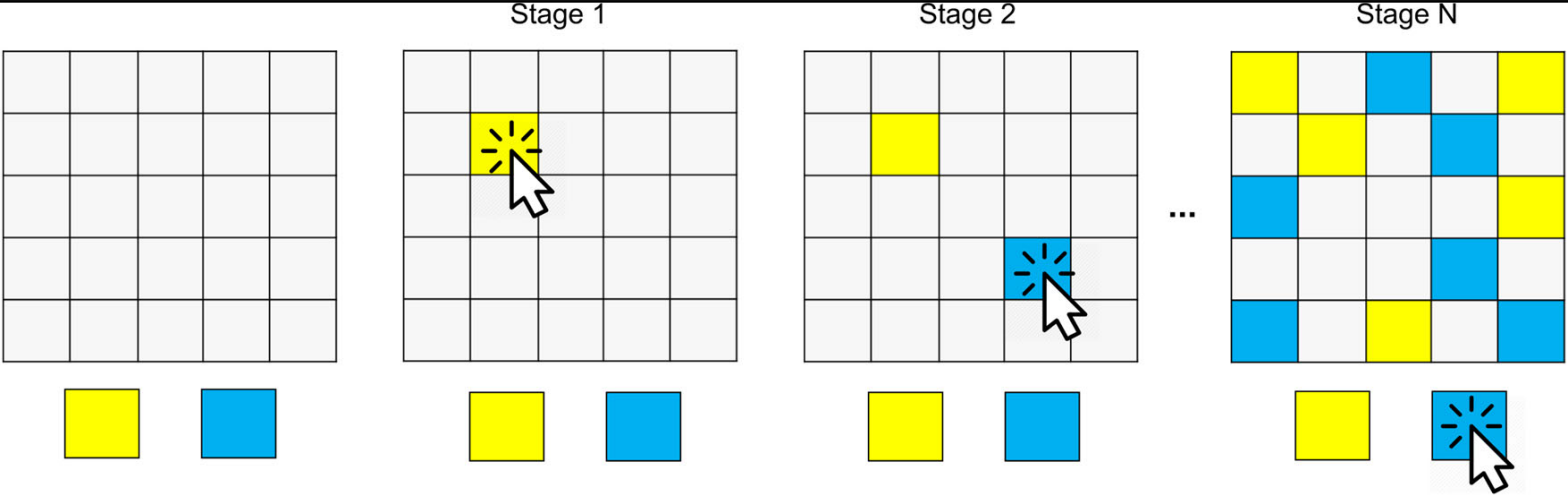
Information gathering task. Subjects were asked to indicate whether the majority of the 25 covered cards (left panel) were of yellow or blue colour. In each game, they were allowed to uncover as many cards as they wished (middle panels) by clicking on a covered card. When they had decided they indicated their decision by selecting the respective colour (right panel)

The subjects performed a paradigm based on the ‘information sampling task’^7,11,31,32^ (Fig. 1). In each game, subjects saw 25 covered cards (depicted by gray squares). Each of these cards could be uncovered using the computer mouse, to reveal one of two colours. In every game subjects were instructed to indicate whether they considered the majority of cards to be blue or yellow (the colours actually varied between games, the nomenclature here is used for simplicity). They were allowed to uncover as many cards as needed (a short delay of 250 ms was introduced between opening the tiles), without restriction on the time spent on the task. Once they felt ‘certain enough’ (detailed instructions are provided in Supplementary Information) they indicated their final decision by selecting the respective colour. After their decision, a short feedback screen (1000 ms) provided information about how many points were won (current and total points), and the next game followed immediately. Subjects had to open at least one card prior to deciding, but there was no maximum, so that subjects were allowed to sample all 25 cards possible before deciding.

We implemented two different reward schemes (the order of presentation of the schemes was kept constant across subjects). In the first set of 10 games (‘fixed’ condition), subjects won or lost 100 points by declaring the correct or incorrect colour, irrespective of the number of cards they uncovered. Then, in the second set of 10 games (‘decreasing’ condition), the potential win decreased as a function of sampling. The potential win started at 250 points and decreased by 10 points for every card that was uncovered (e.g., a correct decision after 4 opened cards would provide 250-4*10 = 210 points). Subjects lost 100 points for a wrong decision, irrespective of the number of uncovered cards. Before the start of the fixed condition, subjects performed one practice game to familiarise themselves with the task. Details about the sequences shown are provided in the Supplementary Information.

### Behavioural analysis

We analysed performance in this task using repeated-measures ANOVAs with within-subject factor condition (fixed, decreasing) and between-subject factor group (high, low compulsives). These analyses were complemented by independent-sample *t*-tests.

Based on previous findings that patients with OCD sampled more in the fixed condition and won more points^7^, a priori we focused our analyses on performance differences (esp. number of draws, points won) between the groups in the fixed condition. In addition, we explored how information gathering in the fixed condition was related to a questionnaire-based report of intolerance of uncertainty^33^ and whether this additionally explains information gathering, over and above the compulsivity group. Effect size metrics (partial eta-squared *η*_*p*_^*2*^, Cohen’s d) and 95% confidence intervals (CI) are reported where appropriate.

### Computational modelling

To understand the cognitive processes that were driving the behavioural differences, we fitted a previously developed Bayesian computational model^7,34^. A description of the model, the data fitting procedure and model comparison can be found in the supplementary material. In the best-fitting model, subjects form a (Bayesian) belief about which of the colours is more likely to form a majority based on the cards they have unveiled so far (i.e., ‘how likely is it that there are 13 or more yellow cards?’). Subjects then use this belief to arbitrate whether to sample more cards (non-deciding) or to decide in favour of one of the two colours. This arbitration is formed by computing state-action values (*Q*-values)^35^ that indicate the worth of taking each possible action. The *Q*-values for choosing the colours are computed based on the belief about whether a particular colour forms a majority, weighted by the outcomes of choosing the (in-)correct colour. The *Q*-value for non-deciding consists of two factors. One is the belief about how certain one will be if one continues with sampling (i.e., weighted *Q*-values of future states, computed using backwards induction). The second factor is a subjective cost per step (or urgency signal^36–39^) that promotes earlier decisions. The ultimate arbitration between these three *Q*-values is determined by a softmax choice rule^40^ with an additional lapse rate^41^.

**Figure 2:**
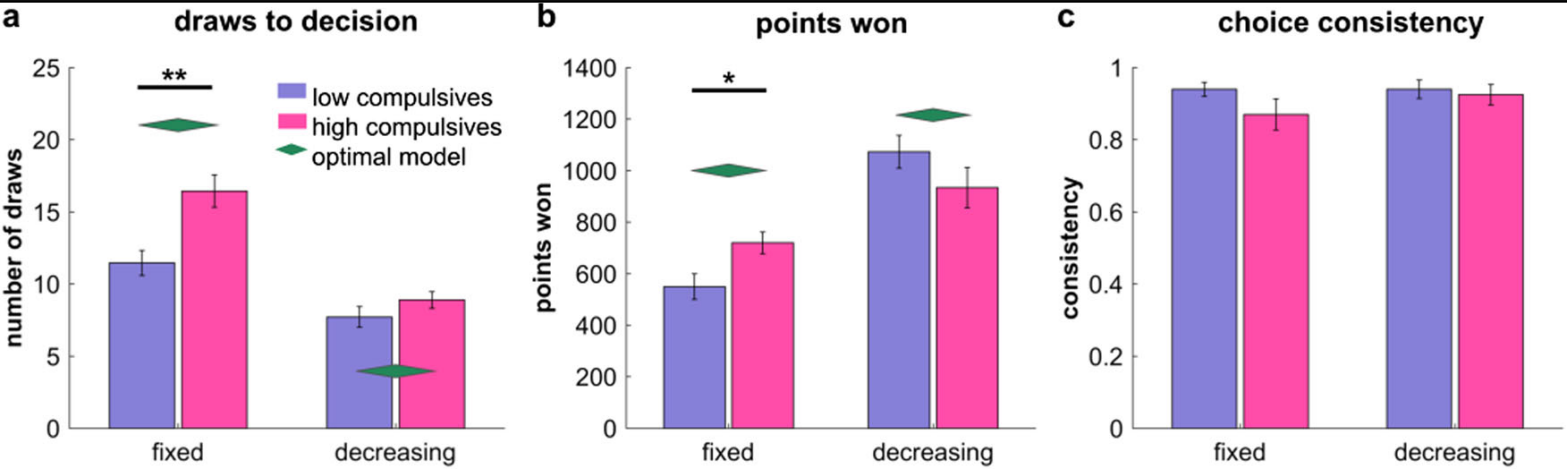
High compulsives sample more information when no external costs apply. **a** High compulsive subjects gather more information before making a decision in the fixed condition, where sampling came at no external costs. **b** This increased information gathering led to higher winnings in the fixed condition, with no difference evident in the decreasing condition. **c** Subjects did not differ in their choice consistency, i.e., whether they picked the colour that was more plentiful at the time of choice. Computational modelling revealed that performance high compulsive subjects was more similar to an optimal agent (green diamonds) in the fixed condition, whereas in the decreasing condition, the low compulsive subjects were slightly more optimal in turns of draws and points won. **p < .01, *p < .05

In the model comparison (cf Supplementary Information), we found that the subjective costs per step followed a nonlinear, sigmoidal, function. This means the subjective costs of sampling were small initially but increased markedly as more information was gathered, similar to a previously described urgency signal^36^. This process is mainly controlled by an ‘impatience’ parameter *p* that describes the stage at which these costs start escalating. The model comparison also revealed that subjects did not correctly represent the external costs in the decreasing condition, leading to suboptimal oversampling in this condition (cf Fig. 2, Supplementary Information). This is why the winning model absorbs both external and internal costs in the urgency signal.

### Model-based analyses

To examine the cognitive mechanisms behind the group differences we fitted a set of recently developed models to each subject’s data (computational model is detailed in Supplementary Information). Using the best fitting model (determined using Bayesian Information Criterion), we then compared the model predictions between the two groups. First, we compared decision thresholds so as to formalize any variation in information gathering behaviour. Decision thresholds are well known from passive evidence accumulation models that use a fixed stopping rule^42,43^. At each stage of information gathering, these thresholds characterise the mean difference in the evidence for the two colours at which subjects are willing to make a decision. In our task the threshold is influenced by the finiteness of the problem (i.e., that there are only 25 squares to sample and a majority of 13 cards is sufficient to be 100% certain) and the subjective cost of sampling, which reduces the *Q*-values for non-deciding (cf. Fig. S3). We used our computational model to compute the decision thresholds, computed as the mean evidence difference when a simulated agent (using the best-fitting subject-specific parameters) made a decision, separated for each sampling stage. With this measure we can assess how much evidence a (simulated) subject needs at each stage^39^, but also examine how a decision criterion might collapse as a function of sampling.

In our task, the collapsing decision threshold is driven by two factors: the finite horizon of the task, and a subjective urgency signal. The former factor is independent of subjects’ preferences and captures the fact that as one gets close to opening all cards, a smaller evidence difference is needed to reach an absolute majority (i.e., 13 cards). The second factor, which we term urgency, is based on the observation that subjects express subjective costs of sampling information and these costs escalate as sampling proceeds. The stage when these costs escalate is determined by the impatience parameter *p* (midpoint of a sigmoid cost function).

Group differences for decision thresholds and urgency signals were assessed using a cluster-extent permutation test (*p* < .05, height threshold *t* = 1, 1000 iterations)^44^.

To understand which aspects of the model were giving rise to the observed differences, we compared model parameters between groups using non-parametric Wilcoxon rank-sum tests, focusing on the impatience parameter *p* in the fixed condition, which our previous work suggested was diagnostic for patients with OCD^7^.

## Results

### Increased information gathering and winnings in high compulsive subjects

We first asked whether subjects with high compulsive scores sought more information before making decisions by comparing the number of cards they opened prior to a decision. We found a main effect of group (*F*(1,38) = 10.15, *p* = .003, *η*_*p*_^2^ = .211 Fig. 2a) showing that high compulsives gathered more information before deciding, relative to the low compulsive group. An additional interaction effect (*F*(1,38) = 7.45, *p* = .010, *η*_*p*_^2^ = .164) demonstrates that the group difference was stronger in the fixed condition, in which no external cost for sampling was imposed, as confirmed by a significant effect in this condition (*t*(38) = 3.52, *p* = .001, d = 1.11, 95% confidence intervals CI: 2.1–7.9) but not in the decreasing condition (*t*(38) = 1.28, *p* = .207, d = .40, CI: −.68−3.03) in which costs were imposed. The effect in the fixed condition also remained when adding depression (BDI total), anxiety (STAI trait and state) and IQ scores as covariates (multiple regression analysis, *t*(34) = 3.26, *p* = .003), thus ensuring that the difference was driven by the compulsivity characteristics of the groups. We also found that both groups gathered more information in the fixed condition than in the decreasing condition, evident in a main effect of condition (*F*(1,38) = 65.38, *p* < .001, *η*_*p*_^2^ = 0.632).

We next asked whether this increased sampling behaviour had a beneficial effect on outcomes, i.e. whether the high compulsive group won more points. A significant group-by-condition interaction (*F*(1,38) = 7.00, *p* = .012, *η*_*p*_^2^ = .156, Fig. 2b), but no group main effect (*F*(1,38) = .063, *p* = .803, *η*_*p*_^2^ = .002) indicated that subjects with high compulsivity scores won more points. However, this was only the case in the fixed condition, as confirmed in a direct comparison (fixed condition: *t*(38) = 2.60, *p* = .013, d = .82, CI: 37−302; decreasing condition: *t*(38) = 1.38, *p* = .176, d = .44, CI: −65–343). Again, the effect in the fixed condition remained when controlling for anxiety, depressive and IQ scores (*t*(34) = 2.71, *p* = .011). Additionally, a main effect of condition (*F*(1,38) = 39.93, *p* < .001, *η*_*p*_^2^ = .512) showed that both groups won more points in the decreasing condition, as would an optimal performing agent (cf. Fig. 2b).

To confirm that improved winnings reflected increased sampling, and not just less random behaviour, we examined whether subjects selected the colour that were more plentiful at the time of the decision (here termed choice consistency). Note that this does not directly translate into whether the subjects chose the colour that formed the overall majority, which in turn is directly reflected in the subjects’ winnings. We found no evidence for a group difference (*F*(1,38) = 1.78, *p* = .190, *η*_*p*_^2^ = .045, Fig. 2c), nor a condition (*F*(1,38) = .920, *p* = .344, *η*_*p*_^2^ = .024) or interaction effect (*F*(1,38) = .920, *p* = .344, *η*_*p*_^2^ = .024). This indicates similar levels of randomness in both groups.

### Independent effects of intolerance of uncertainty and compulsivity on information gathering

On a symptom level, excessive information gathering and indecisiveness have often been related to an intolerance of uncertainty and a pervasive perfectionism, especially in the context of OCD^5,45^. We thus obtained the subjects’ self-reported intolerance of uncertainty (IU questionnaire^33^ total score) and perfectionism (Frost Multidimensional Perfectionism Scale total; FMPS^46^). We observed a significant group difference in their intolerance of uncertainty (*t*(38) = −2.05, *p* = .047; Table 1), but not in perfectionism (*t*(38) = -.94, *p* = .353; Table 1). To assess whether IU and compulsivity independently explained the increased information gathering behaviour in the fixed condition, we assessed their independent contributions using a multiple regression. We found that both compulsivity (*t*(37) = 2.80, *p* = .008) and self-reported intolerance of uncertainty (*t*(37) = 2.30, *p* = .027) predicted information gathering behaviour, independently of each other (Fig. S6). No other measure (anxiety, depression, perfectionism, impulsivity, age, IQ) correlated with information gathering in the fixed condition (all *p*’s > .2).

### Information gathering along the compulsivity spectrum

**Figure 3:**
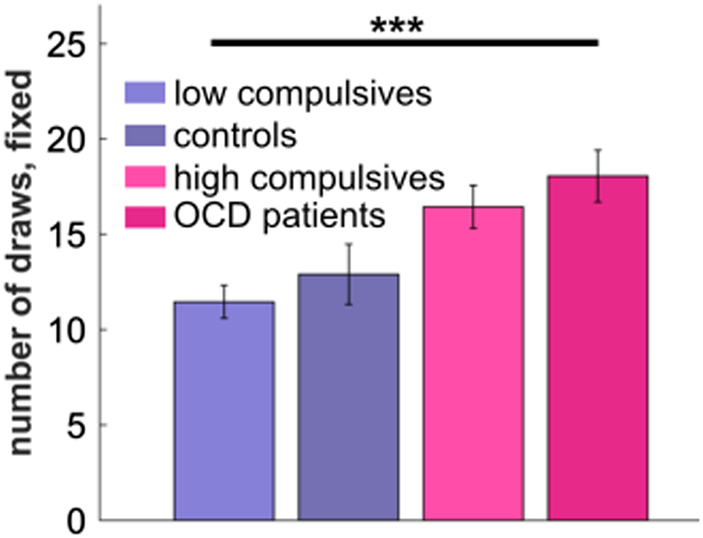
Information gathering as a marker for a compulsivity spectrum. Pooling data from two studies with OCD patients and non-clinical high compulsives confirms a linear increase of information gathering in the fixed condition along compulsivity spectrum. ***p < .001

Our finding of increased information gathering in the fixed condition in high compulsive subjects closely resembles our previous finding in adolescent OCD patients^7^. A key prediction of a conception of compulsivity as a dimension would be that information gathering increases along the compulsivity spectrum, and where OCD patients would express the most extreme information gathering behaviour. We examined this prediction by pooling the two studies (previous OCD study and present study) and tested for a linear increase in information gathering across groups (low compulsives < controls in OCD study [not selected for low compulsivity] < high compulsives < OCD patients). Here we found a highly significant effect of compulsivity group on information gathering (Fig. 3, *t*(70) = 4.33, *p* < .001), supporting the notion that information gathering is a general feature of a compulsivity spectrum.

### Increased decision threshold in high compulsives due to altered subjective urgency in fixed condition

To understand the mechanisms and processes likely to drive the observed group difference in information gathering, we used a Bayesian computational model. Model comparison (Fig. S1) revealed that task behaviour of subjects was best described if we assume an urgency signal that rises in a nonlinearly manner during sampling and promotes choosing, independently of current evidence. This is implemented in our model in terms of subjective costs of sampling that escalate from an initially small value as sampling progresses, closely resembling urgency signals proposed for perceptual decision making^36,37,47^.

**Figure 4:**
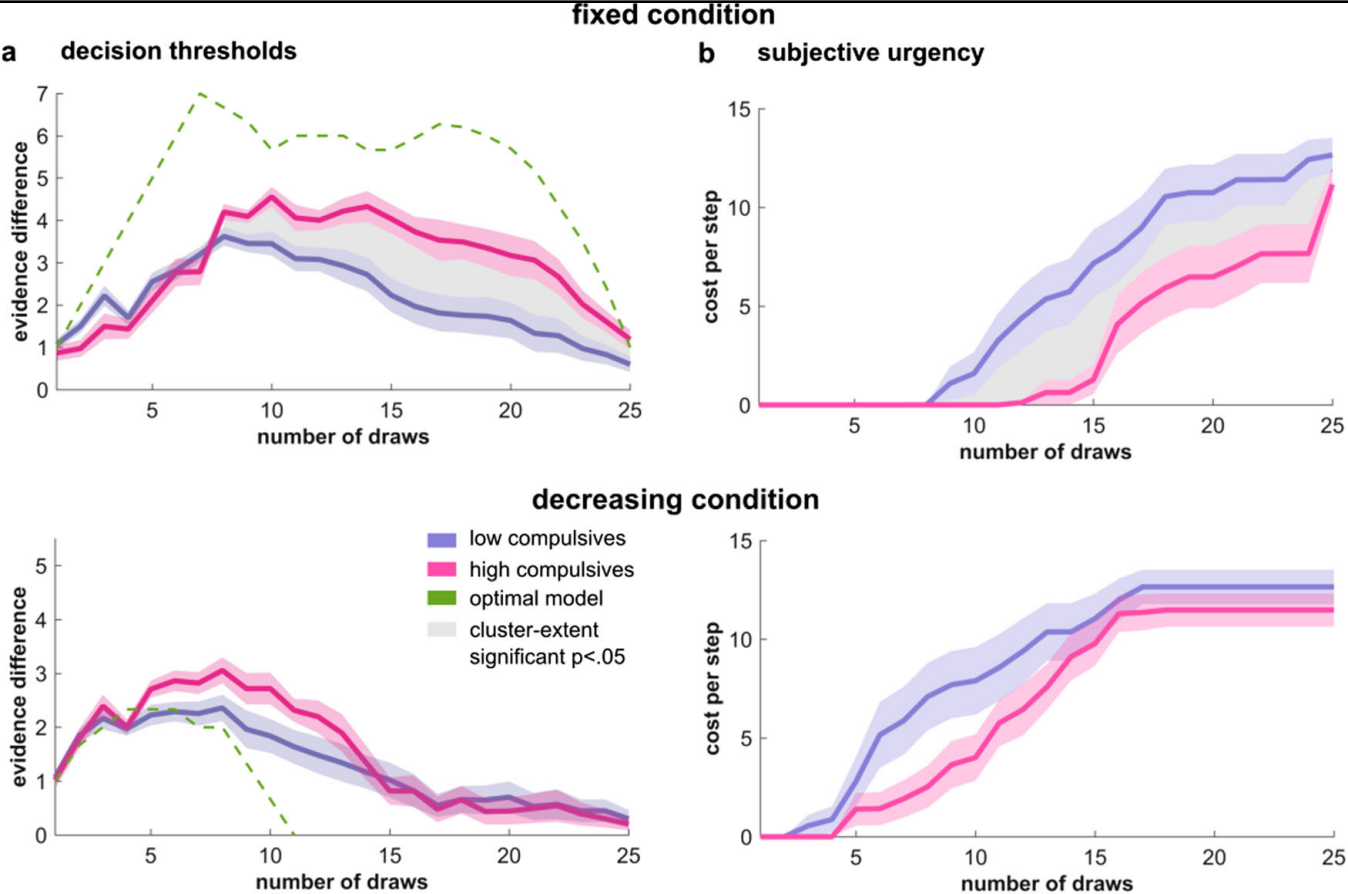
Increased decision thresholds and delayed urgency signals in the high compulsive group in fixed condition. **a** Decision thresholds indicate the difference in evidence needed for making a decision. The thresholds collapse as a function of sampling, in line with an increasing urgency signal found in perceptual decision making^36, 39^. Importantly, the decision threshold in the fixed condition collapses significantly earlier in the low compulsive group, indicating that high compulsive subjects maintain a higher decision criterion for longer and consequently sample more information before making a decision. Decision threshold differences in the decreasing condition did not reach significance (lower panel). Green lines indicates decision thresholds of an optimal model. The initial rise of the thresholds reflects the maximal evidence difference possible at this stage (e.g., maximal evidence difference at Stage 2 is 2). **b** The urgency to choose, here cast as subjective costs for sampling, for the high compulsive group escalates later in the fixed condition. This is apparent in the fixed condition (upper panel) from the 10^th^ draw onwards, but not in the decreasing condition (lower panel)

To formalize, and assess, how high and low compulsive subjects differ in their model predictions, we computed decision thresholds which reflected how much more evidence for one colour is needed to make a decision at each stage in the information sampling process (i.e., number of opened yellow—blue cards). Using model simulations, we found these thresholds collapsed over samples, in other words subjects became more liberal and were content to make a decision on weaker grounds. This collapse occurred significantly earlier for low compared to the high compulsive group in the fixed (Fig. 4a; *p* = .024, cluster-extent correction), but not in the decreasing, condition (*p* = .077, cluster-extent correction).

One of the key drivers of the collapsing decision thresholds is an urgency signal, and in our model this is cast as subjective costs per step. In the fixed condition there are no extrinsic cost, so this urgency reflects purely intrinsic (energy, cognitive or time) costs for continuing to sample. In the decreasing condition, the best-fitting model encompasses the externally applied costs within a single term (see Supplementary Information for detailed analyses of different costing models), which is why these costs arise markedly earlier in the latter condition (Fig. 4b).

When analysing this urgency signal we found that it arose significantly earlier in the low compared to the high compulsive subjects in the fixed condition (Fig. 4b; fixed condition: *p* = .004, cluster-extent correction; decreasing condition: *p* = .212, cluster-extent correction).

Finally, we compared model parameters to probe group differences in decision thresholds further. We found a significant difference in the impatience parameter *p* for the fixed condition (*z*(311) = −2.66, *p* = .008, Fig. S2), similar to what we observed in a previous study of diagnosed patients with OCD^7^. This parameter is a key determinant of when the urgency signal starts escalating. We did not observe any difference for other model parameters (cf. Fig. S2).

## Discussion

Humans differ in how they negotiate speed-accuracy tradeoffs^48–50^. In sequential information gathering patients with OCD exhibit symptoms that can be construed as a distortion of this speed-accuracy tradeoff due to overweighting correct decisions^6–13^. Here we use computational modelling of the behaviour of a carefully curated sample of young people to demonstrate that excessive information gathering is a defining feature of an obsessive-compulsive spectrum, going beyond the clinical manifestation of OCD.

A pervasive indecisiveness and intolerance of uncertainty is seen as part of the core of OCD patients’ obsessions and compulsions^4,51^. Such symptoms can be construed as an overweighting of the correctness of decisions, and an excessive investment in information gathering. Although such theories of compulsivity are venerable^52,53^, behavioural analyses that test this idea are relatively scarce. In this study, we extend previous observations with OCD patients^6–13^ by showing that subjects with high, but non-clinical, symptoms of compulsivity exhibit a similar behavioural phenotype, albeit to an attenuated degree. Thus, both patients and non-clinical high compulsives manifest increased information gathering behaviour when there is no external cost for sampling. Notably this information gathering behaviour increased monotonically along a compulsivity spectrum, suggesting that increased information gathering generalises across a spectrum of neurodiversity. This includes, but is not confined to, clinical disorder at its extreme end. Interestingly, this increased sampling led to a better performance for subjects with high compulsivity scores in both studies, suggesting increased information gathering can be task beneficial (a feature rarely observed as consequence of a psychiatric symptom). Indeed, the fact that subjects with high compulsivity scores perform more proficiently on this task raises interesting questions, especially as indecisiveness is thought of as incapacitating^4,5,51^.

We used computational modelling to probe the underlying cognitive mechanisms and revealed that this altered sampling behaviour in the fixed condition was due to an increased decision threshold in high compulsives or, more exactly, a slower collapse of the decision threshold across sampling. That is, the high compulsive group used a more restrictive decision criterion for a longer period. This slower collapse of a decision threshold was captured in our model as a delayed emergence of a subjective urgency to choose. Specifically, the impatience parameter that governs the stage at which this urgency grows most quickly is greater in high compulsive subjects, in accordance with our previous finding in patients with OCD^7^.

It is notable that high compulsives (as well as OCD patients as shown previously^7^) all have collapsing decision thresholds, with the onset of this collapse adapting with external task demands (i.e., for the fixed vs decreasing condition). This suggests that an elevated decision threshold is not an expression of cognitive inflexibility or rigidity, i.e. an inability to adjust strategies to maximise external rewards, but is the result of an arbitration between an internal need for certainty, and other internal and external demands, such as explicit sampling costs. Indeed, we found a group effect for information sampling in the fixed condition alone, and not in the decreasing condition (although differences in the reward structures in the two tasks that could also have contributed to this). The observation that high compulsive subjects had a lower reward rate (number of points per unit time, cf. Supplementary Information for analyses) than low compulsive subjects in the decreasing condition might suggest they were not as sensitive to this implicit metric. However, this reward rate did not differ between groups in the fixed condition. Thus although high compulsive subjects won more points overall, they did not win more points per unit time. This favours an interpretations that compulsive subjects solve a speed-accuracy tradeoff differently, favouring accuracy when there is no explicit costs for extended elaborations.

Considerations of the rate of reward prompt speculation as to whether the urgency signal is more directly linked to the time that has passed, the effort that has so far been exerted^54^, or to the amount of information that has been obtained. As for most other sampling tasks, we lack the sensitivity to distinguish between these linked options. However, teasing these factors apart in future studies could shed light on whether the impatience that we observe is due to a perceived waste of sampling-time, a cognitive capacity issue due to an overwhelming amount of information, or some other additional factors.

Urgency signals to promote liberal decision making as a function of sampling have previously been described in perceptual decision making^36,38^. These signals are assumed to arise nonlinearly^37,47^, as we found, and to modulate evidence accumulation in the brain^36,38,39^. Given the relevance in compulsivity, it is interesting to make conjectures about the neural origins of this signal. One of the key areas described to modulate decision thresholds is the subthalamic nucleus (STN)^55–58^, which has been found to be compromised in OCD^59^ and is a common target for deep brain stimulation in refractory OCD patients^60,61^. It is assumed that the STN closely communicates with areas of the medial prefrontal wall^55,62,63^, such as the anterior cingulate cortex, which also has been found to be impaired in structure and function^64,65^ and is another target area for OCD neurosurgery^66^.

It is interesting to conjecture how increased information gathering is related to other neurocognitive mechanisms that have been found as impaired in compulsivity, such as an excessive habitual behaviour^16,67^. It is possible that cognitive features such as inflexibility or a dominance of habitual modes of behaviour describe separate neurocognitive subtypes that express in similar compulsive symptoms. Alternatively, these features may arise at different stages in the evolution of what is often a chronic disorder, and where our findings pertain to an early phase as seen in our adolescent and young adult samples.

In our study, we observed that increased information gathering in the fixed condition did not load solely on one of the obsessive-compulsive subscales (cf. Supplementary Information). Rather, it loaded to some degree on most of the PI-WSUR subscales. This supports the notion that information gathering is not portraying a subtype on a symptom level (e.g., only compulsions or obsessions), but rather depicts a specific neurocognitive phenotype.

In this study, we used a targeted recruitment approach, where we carefully selected 40 subjects from a large population-based study. With a sample of 20 subjects per group, the group size was relatively modest and this was partly determined by our very selective recruitment and matching approach. We recruited subjects on the basis that they scored either high or low on a compulsivity spectrum, but who were otherwise matched on psychiatric dimensions such as anxiety or depression. This allowed us to study compulsivity independently of other, potentially confounding psychiatric traits and thus enabled us to attribute excessive information gathering more directly to compulsivity. Although modest in size our sample size is in line with previous patient studies that reported similar sample^7–11^ and relatively large effect sizes^7,10^.

Our study provides evidence for increased information gathering in conditions with no external costs, behaviour that is characteristic of a compulsivity spectrum, and that is seen in both non-clinical high compulsive subjects as well as in patients with OCD. This behaviour is driven by elevated decision thresholds and a slower emergence of an urgency to respond. By recruiting from a population-based sample we control for potential confounders, such as other psychiatric dimensions, age or intellectual abilities. The findings lead us to propose that compulsivity is a phenotypic spectrum that is characterised by an increased need for certainty.

## Electronic supplementary material

Supplementary Information
