# Supplementary material for: Increased decision thresholds trigger extended information gathering across the compulsivity spectrum
Source: Transl Psychiatry. 2017 Dec 18;7:1296. doi: 10.1038/s41398-017-0040-3 (PMC5802702; doi:10.1038/s41398-017-0040-3)
Supplement: Supplementary file 1 — Supplementary Information [file 41398_2017_40_MOESM1_ESM.pdf]

Increased decision thresholds trigger extended information gathering across the compulsivity spectrum

Hauser TU, Moutoussis M, NSPN Consortium, Dayan P & Dolan RJ

Supplementary Information

Supplementary task details and instructions

Instructions

Subjects were instructed during one practice game. The instructions were given as follows:

> "I*n this task, you'll have to decide whether the majority of these 25 cards shown here are [colour 1] or [colour 2]. At the beginning, all cards are covered. To open a card, you need to click on it with the mouse. You can open as many cards as you want until you feel certain enough. When you feel certain enough, then you have to click on the coloured card you think is the majority. If you select the correct colour, you will earn [amount] points, otherwise you will lose [amount] points.*"

For the decreasing condition, the cost structure was explained before starting the first costed game:

> "*With every card you open, you will win 10 points less. This time, the potential wins start at 250 points. So if you would decide correctly without opening any card, you'd win 250 points. If you open, let's say 3 cards and you choose the correct colour, then you'd win 220, and so on. However, if you're wrong, you will always lose 100 points.*"

Task sequences

We used similar task sequences as implemented in the information sampling task (IST, CANTAB), and which we used in our previous study on information gathering in OCD patients (Hauser et al., 2017). The sequences were kept the same for all subjects (i.e. the sequence of yellow and blue cards being revealed), but the order of the games was randomised (when a particular sequence is presented in the 10 games of each condition). The sequences used in the IST are all difficult with a generative probability close to 50%. This means that all sequences were challenging and an increase in sampling (esp. in the fixed condition) provided a better estimate about the majority of cards. The sequences used are shown in Fig. S5. Also see Hauser et al. (2017) for a detailed discussion about how the sequences translate into win probabilities.

Winning more or more quickly? Reward rate analysis

In our main analyses, we have shown that high compulsive subjects gather more information in the fixed condition and also win more points. A prominent notion in perceptual decision making is one of reward rates (Balci et al., 2011; Bogacz et al., 2006, 2010) that capitalise on reward per unit time rather than reward per game (as measured here). This raises the question of whether high compulsives would also outperform low compulsives if we hypothetically restrict the total time they spent on performing the task, rather than giving subjects a fixed number of games to play without time restrictions.

We examined whether a reward rate (reward per minute) differed between the two groups and found a significant group-by-condition interaction ($F(1,38)=9.52$, $p=.004$, $\eta_p^2=.20$) in the absence of a group main effect ($F(1,38)=2.15$, $p=.150$, $\eta_p^2=.05$), suggesting that the reward rate differed in only one of the conditions. A post-hoc exploratory group comparison showed no difference between the groups in the fixed condition ($t(38)=.63$, $p=.534$, $d=.19$, CI: -73-138, Fig. S7A), mainly due to fact that increased winnings in high compulsive subjects were levelled out by a slightly longer (although not significantly so, $t(38)=1.39$, $p=.172$, $d=.45$, CI: -11-60, Fig. S7B) time on task. This suggests that despite high compulsive subjects winning more overall, they might not have done so under a time-dependent reimbursement scheme. Instead, we found a significant group difference for the reward rate in the decreasing condition ($t(38)=2.27$, $p=.029$, $d=.72$, CI:24-453, Fig. S7A) with high compulsive subjects having a lower reward rate. This was because they spent marginally more time on that task ($t(38)=1.69$, $p=.099$, $d=.53$, CI: -4.3-47.5, Fig. S7B), without earning more points (Fig. 2B). A similar result was obtained when we used a reward rate per sample rather than time (fixed condition: low compulsives: 4.97±1.83; high compulsives: 4.62±1.49; $t(38)=.67$, $p=.505$; decreasing condition: low compulsives: 15.76±6.50; high compulsives: 10.75±4.39; $t(38)=2.86$, $p=.007$).

Lastly, we explored whether the two groups differed in their reaction times for uncovering each card. A main effect of condition ($F(1,38)=21.19$, $p<.001$, $\eta_p^2=.36$, Fig. S7C) revealed that both groups spent more time per draw during the decreasing condition, but there was no significant difference between the groups (main effect of group $F(1,38)=.03$, $p=.856$, $\eta_p^2=.001$; group-by-condition interaction $F(1,38)=3.80$, $p=.059$, $\eta_p^2=.091$).

Information gathering and obsessive-compulsive symptoms

In our paper, we directly compared high and low compulsive subjects. However, we also conjectured that it would be interesting to examine post-hoc whether some obsessive-compulsive symptom dimensions were more closely related than others.

We thus analysed the subscales (across both groups) of the PI-WSUR (Burns et al., 1996), our measure of obsessive-compulsive symptoms. There are five different subscales of this questionnaire: obsessional thoughts about harm to oneself or others; obsessional impulses to harm oneself or others; contamination obsessions and washing compulsions; checking compulsions; dressing/grooming compulsions.

We found that information gathering (number of draws) in the fixed condition most strongly correlate with the total score ($r=.45$, $p=.004$). They also correlate highly with the checking compulsions ($r=.44$, $p=.004$), a contamination dimension ($r=.41$, $p=.009$) and dressing compulsions ($r=.41$, $p=.009$). Information gathering showed a weaker association with obsessional thought about harm ($r=.33$, $p=.037$), and did not correlate with the impulses to harm ($r=.01$, $p=.936$). These findings suggest that information gathering is not a specific marker for a specific symptomatology, but rather obsessive-compulsive symptoms in general.

Computational model for information gathering task

We previously developed a computational model for given information gathering task (Hauser et al., 2017). In this study, we used the same model to make inference about the computational mechanisms involved in this task. Here, we repeat a description of the model for completeness, taken from Hauser et al. (2017).

Our model assumes that subjects try to infer the colour that forms the majority of cards, based on the cards seen so far. This means that the subjects infer the probability that the majority of cards belongs to a particular colour, e.g. yellow (*y*):

$$P(MY \mid n_y, N) \tag{1.1}$$

where *MY* depicts the majority of yellow cards, n_y the number of opened yellow cards, and *N* the total number of opened cards.

P(MY) is fully determined as soon as 13 or more of the opened cards belong to one colour (of a total number of cards: N_{tot}=25). Otherwise, the probability has to be inferred:

$$P(MY \mid n_y, N) = \begin{Bmatrix} if\ n_y > N_{tot}/2; = 1 \\ if\ N - n_y > N_{tot}/2; = 0 \\ else\ eq.1.3 \end{Bmatrix} \tag{1.2}$$

This can be inferred by calculating the probability of the majority of cards being yellow, given a specific generative probability *q* (proportion of yellow and blue cards in the machinery that produces the sequence), weighted by the likelihood of this generative probability based on the currently seen cards:

$$\begin{aligned} P(MY \mid n_y, N) &= \int_0^1 P(MY \mid q, n_y, N) P(q \mid n_y, N) dq \\ &= \sum_{Y > N_{tot}/2}^{N_{tot}-(N-n_y)} \int_0^1 P(Y, N_{tot} \mid q, n_y, N) P(q \mid n_y, N) dq \\ &= \sum_{Y > N_{tot}/2}^{N_{tot}-(N-n_y)} \int_0^1 P(Y - n_y, N_{tot} - N \mid q, n_y, N) P(q \mid n_y, N) dq \\ &= \sum_{Y > N_{tot}/2}^{N_{tot}-(N-n_y)} \int_0^1 Bin(Y - n_y \mid q, N_{tot} - N) P(q \mid n_y, N) dq \end{aligned} \tag{1.3}$$

The first expression is a binomial of getting Y-n_y yellow draws out of N_{tot}-N draws (cf eq. (1.5)), given generative probability *q*. The second expression is the probability of the *q* being the generative probability. This can be calculated as follows:

$$\begin{aligned} dqP(q \mid n_y, N) &= \frac{P(n_y \mid q, N) P_0(q \mid N)}{P(n_y \mid N)} dq \\ &= \frac{P(n_y \mid q, N) P_0(q)}{\int P(n_y \mid q', N) P_0(q') dq'} dq \end{aligned} \tag{1.4}$$

We assume that the probability of *n* yellow draws of *N* total draws follows a binomial distribution and that the prior belief about the generating *q* follows a beta distribution (conjugate prior) with the parameters α and β (using α=1, β=1):

$$P(n_y \mid q, N) = \binom{N}{n_y} q^{n_y} (1-q)^{N-n_y} = Bin(n_y \mid N, q) \tag{1.5}$$

$$P_0(q) = Beta(q \mid \alpha, \beta) \tag{1.6}$$

Thus, the posterior can be expressed as (Bishop, 2007):

$$\begin{aligned} & P(q \mid n_y, N)dq \propto \\ & P(n_y \mid q, N)P_0(q)dq = Bin(n_y \mid N, q)Beta(q \mid \alpha, \beta)dq \\ & = Beta(q \mid \alpha + n_y, \beta + (N - n_y))dq \end{aligned} \tag{1.7}$$

The beliefs about the majority of cards are subsequently translated into action-values. The action value of choosing *Y*, (*Q(Y)*), is the product of reward/cost of choosing the right or wrong option (R_{cor}, R_{inc}) and the success-probabilities of these actions. *Q(B)* is calculated analogously.

$$Q(Y \mid n_y, N) = R_{cor}P(MY \mid n_y, N) + R_{inc}P(MB \mid n_b, N) \tag{1.8}$$

The rewards of correctly (R_{cor}) and incorrectly declaring (R_{inc}) can be cast in different ways. According to the objective instructions, in the fixed condition R_{cor} is set to 100 and R_{inc} to -100. For the decreasing condition, we compared two different formulations. In our main model ('subjective'), we also kept R_{cor} constant in the decreasing condition. This was done so that the subjective costs (c_s, cf below) soak up the subjectively perceived overall costs, i.e. a combination of externally imposed and internally generated costs. This way, we can investigate the subjectively perceived costs. Alternatively, we formulated an 'objective' costs model, where R_{cor} changes as a function of step (250, 240, 230, …), as set up in the task. This 'objective' model ('objective linear/nonlinear costs' model in Fig. S1) only differed in the decreasing, but not in the fixed, condition. Additionally, R_{inc} was kept at -100 for all models and conditions.

The action value of not deciding (*Q(ND)*) computes the value of future states in terms of the future action values and their probabilities. Additionally, a cost per step is imposed that assumes that there are internal (and external) costs that emerge when continuing with sampling. *Q(ND)* is calculated using backward induction to solve the Bellman equation, using state values *V(s')* and a cost per step c_s:

$$V(s') = \sum_{\text{all available } a \text{ in } s'} \pi(a, s')Q(a, s') \tag{1.9}$$

$$\begin{aligned} Q(ND \mid n_y, N) &= -c_s + \sum_{\text{all } s'} P(s' \mid n_y, N)V(s') \\ &= -c_s + \sum_{s' = \left\{ \begin{smallmatrix} n_y + i \\ N+1 \end{smallmatrix} \right\}}^{i=[0,1]} P(s' \mid n_y, N)V(s') \end{aligned} \tag{1.10}$$

The probability of reaching state *s'* and seeing *i* new yellow items is based on the current belief state, which in turn is mainly determined by the current evidence n_y, *N*. Thus:

$$\begin{aligned} P(s' \mid n_y, N) &= P(n_y + i, N + 1 \mid n_y, N) \\ &= \int_{\text{all } q} P(q, n_y + i, N + 1 \mid n_y, N)dq \\ &= \int_{\text{all } q} Bin(i \mid 1, q)P(q \mid n_y, N)dq \end{aligned} \tag{1.11}$$

The choice policy π (Fig. S3) for the state-action space is specified as the following softmax function with decision temperature parameter τ and irreducible noise (lapse rate) parameter ξ (e.g., Guitart-Masip et al., 2012):

$$\pi(ND \mid n_y, N) = \frac{e^{Q(ND|n_y,N)/\tau}}{e^{Q(Y|n_y,N)/\tau} + e^{Q(B|n_y,N)/\tau} + e^{Q(ND|n_y,N)/\tau}}(1-\xi) + \frac{\xi}{3} \tag{1.12}$$

Nonlinear sigmoidal cost function

We compared two different possible cost functions: fixed cost per sample vs a model in which the costs per sample increased according to a sigmoid function. The latter could capture the possibility that subjects felt an increasing urgency (Cisek et al., 2009) to decide, for instance if it becomes increasingly annoying to gather more samples with potentially little informational content and waste time, similar to previous reports that show that costs increase nonlinearly (Drugowitsch et al., 2012).

We implemented the nonlinear cost function as a sigmoid, where the cost per step c_s (eq. (1.10)) changes on each step n (1, …, 25):

$$c_s = \frac{c}{1+e^{-k(n-p)}} \tag{1.13}$$

c depicts a scaling factor of all costs. The parameter k denotes the slope of the increase in costs, whereas the patience parameter p describes the midpoint, i.e. at what stage in the game the agent becomes impatient.

The costs parameters were modelled independently for both conditions, i.e. the parameters could soak-up a condition-dependent difference in subjective costs. Model comparison (cf below) revealed that the condition was primarily driving the midpoint p, whereas the slope k and scale parameters c could be shared across conditions. It is important to note that in the winning 'subjective' model, the internal as well as the external costs were conglomerated in this cost module. Albeit inadequately reflecting the instructions in the decreasing condition (linear 10 points less win per draw), model comparison revealed that this model outperformed an 'objective' model, in which the reduction in wins was explicitly modelled by a reduction of R_{cor} as a function of sampling.

Optimal agent simulations

To understand how one would perform this task optimally, we used the model to perform this task. As an optimal agent would have no implicit sampling costs, but a correct representation of the external costs, we used the model with the 'objective' cost structure. Moreover, the subjective costs were set to 0 and the decision temperature τ was set to 1. We used the same sequences as the subjects played with and found that the optimal agent made 21 draws in the fixed and 4 draws in the decreasing condition. This means that it sampled more than our subjects in the fixed, but less in the decreasing condition. This led to a better performance in both conditions with maximal points in the fixed condition (i.e. 1000 points) and 1215 points in the decreasing condition.

Model fitting, model comparison and parameter comparison

Methods

We used maximum LogLikelihood to optimize each subjects' the model parameters. We used a genetic algorithm implemented in Matlab to find global minimum (Goldberg, 1989). We subsequently performed a model comparison using summed AIC (Akaike, 1973) and BIC (Schwarz, 1978). The best model was then used to compare parameters and the model predictions between the two compulsivity groups (cf Fig. S2, main text), and was shown to reproduce the group differences (Fig. S4).

Model comparison

Similar to our previous studies, we compared different variants of our model to reconcile on the best-fitting version. In particular, we investigated how costs of sampling arise. The main comparison was performed between a linear cost model, where the costs of sampling were the same for each step, and a nonlinear cost model, in which the costs escalated over sampling. Similar to our previous paper (Hauser et al., 2017), we found that a

nonlinear cost function clearly outperformed the linear variant (Fig. S1 for all model comparison results). We then tested whether some parameters were shared between the fixed and decreasing condition. We found that a model that shared the cost-scaling parameter c and the slope k, but not the impatience parameter p performed best. This means that the conditions differ when the subjects become impatient (i.e. the costs escalate), but not by how much. We additionally found that there was no improvement in model fit if we use separate decision temperatures (τ) for the conditions. Also, an implementation of 'objective' costs (cf above) performed worse. Lastly, we compared our model to a version in which subjects made inference about the generative probability ($M_{generative}$) rather than the actual majority of cards (cf. Hauser et al., 2017). This model performed only minimally worse than the winning model (mean BIC difference per subject $\Delta BIC = .70$) and all the model findings and group differences reported in this paper replicated with this model (data not shown).

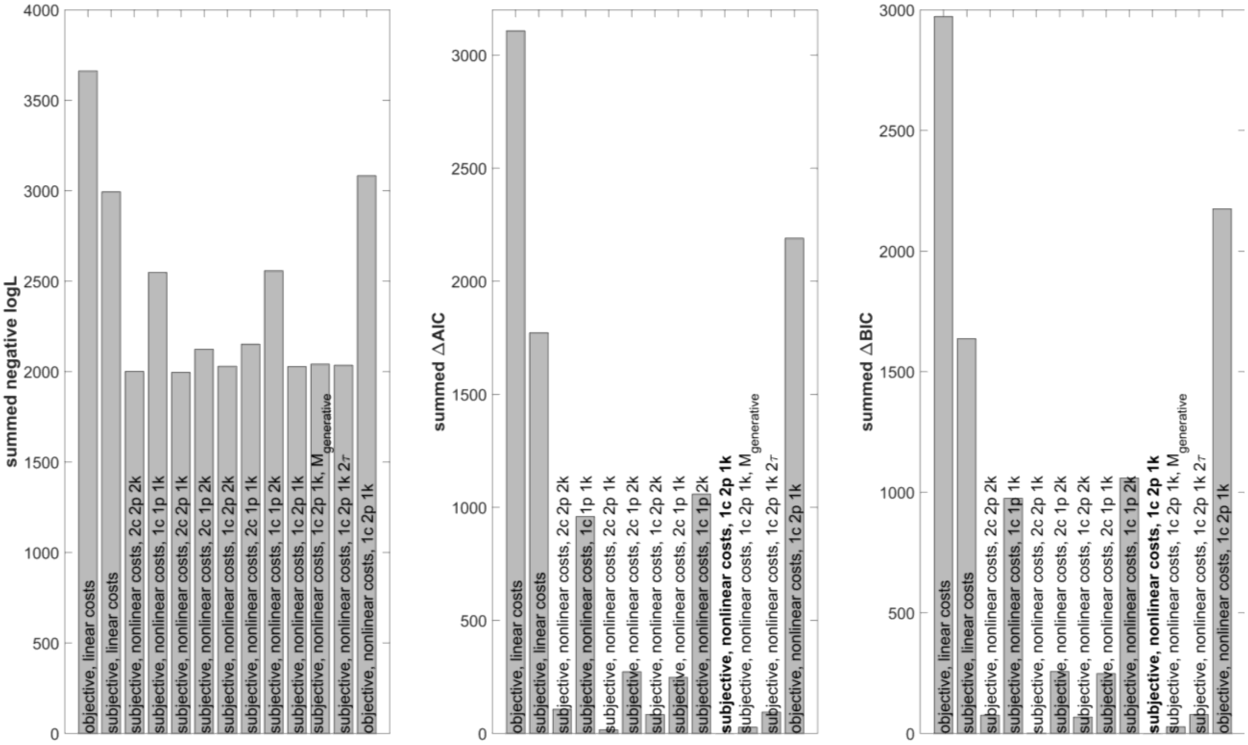

Figure S1: Model comparison information gathering task. Model comparison reveals that subjects' behaviour is described best by a model that contains a subjective, nonlinear representation of sampling costs. The best fitting model has one cost scaling parameter c and one cost slope k, but two cost midpoints p (one for each condition). There was no benefit for having two separate temperature parameters τ. A model that inferred about the generative probability ($M_{generative}$) rather than the majority, performed highly similar. See main text and supplement above for further explanations. Best fitting model is shown in bold, model performance shown relative to best model. ΔAIC: difference in Akaike information criterion (compared to best model), ΔBIC: Bayesian information criterion difference, logL: logLikelihood.

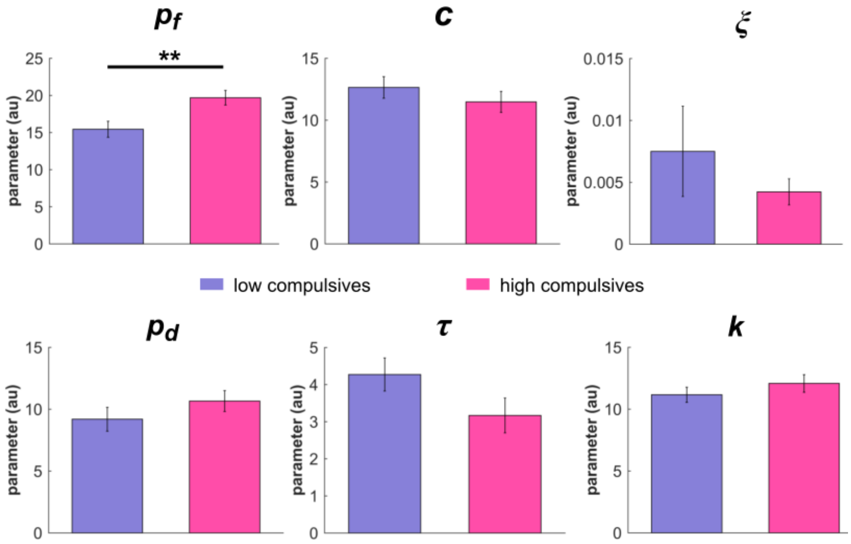

Figure S2: Model parameter comparison. Comparison of the model parameters reveals that the groups in their impatience parameter for the fixed condition p_f. This parameter indicates after how many samples a subject becomes impatient and becomes more liberal in the decision making, and has also been found to be increased in a previous study with OCD patients (Hauser et al., 2017). None of the other parameters differed between groups. ** p<.01; $_f$: fixed condition; $_d$: decreasing condition.

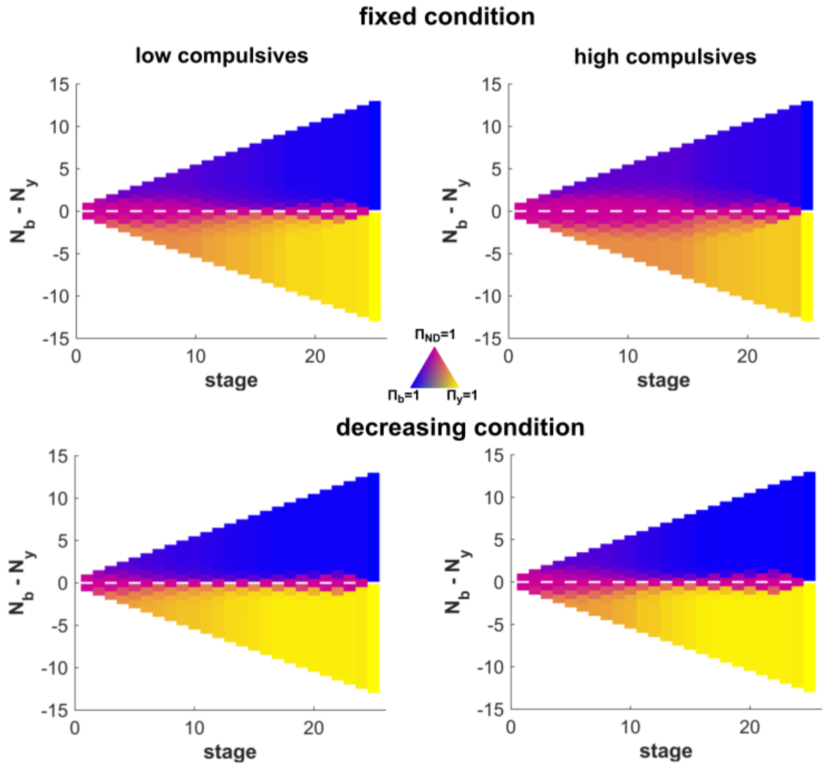

Figure S3. Decision policy of the computational model. The model policy describes the probability of making a specific action (deciding for yellow π_y, deciding for blue π_b, or continuing sampling π_{ND}) at each possible state (x axis: number of cards opened in total, y axis: difference between number of blue and yellow items opened). This policy plot reveals that both groups were generally more willing to decide earlier in the decreasing condition (lower vs. upper panels: smaller pink areas). For the fixed condition, the high compulsive subjects (upper right) were more likely to sample for longer (extended pink area) than was the case for low compulsive subjects (upper left).

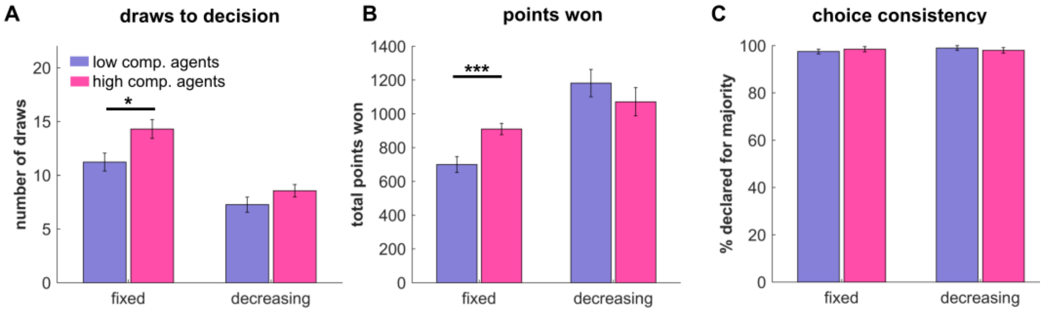

Figure S4. Simulated behaviour. Using the best-fitting parameters of the winning model, we simulated behaviour to test whether we can re-generate the behavioural differences between the groups. Indeed, the model showed significantly increased earnings in the fixed condition for the high compulsive agents (B) as well as an increase in the number of samples before decision (A). No difference was observes in the choice accuracy (C), in line with the behavioural data. * p<.05, *** p<.001

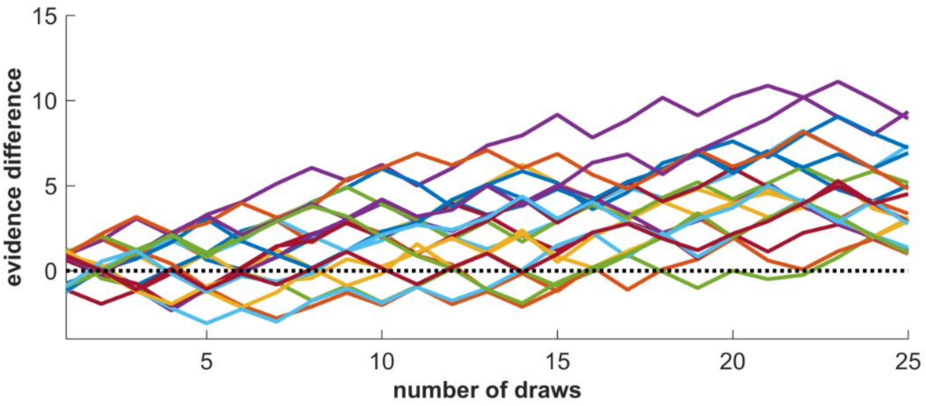

Figure S5. Sequences used for study. Sequences presented to subjects were the same as in Hauser et al. (2017). Each sequence is shown in a different colour (plus some random noise to better visualise sequences) and the indifference line (no evidence in favour of any colour) is displayed as a dotted line.

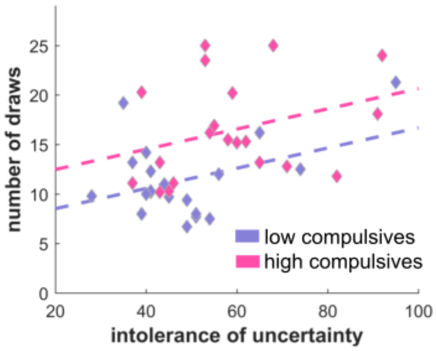

Figure S6. Independent contributions of compulsivity and intolerance of uncertainty to information gathering. Information gathering behaviour in the fixed condition was influenced by two factors: the compulsivity group with high compulsives (pink) sampling more than low compulsives (violet). The other factor was their self-

reported intolerance of uncertainty (x-axis). Both factors independently predicted information gathering (lines: predictions of regression model).

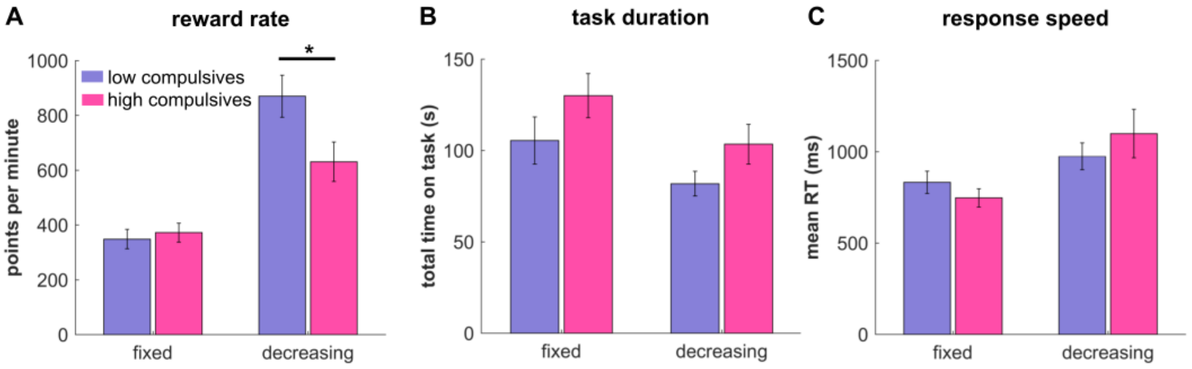

Figure S7. Reward rate in high and low compulsive subjects. Although the high compulsive subjects won significantly more points in the fixed condition, their relative wins per unit time did not differ significantly from low compulsive subjects (A). This means that high compulsive subjects sacrificed time for accuracy, implying that they placed greater weight on making correct decisions than on speed. Conversely, they had a significantly lower reward rate in the decreasing condition, because they spent marginally more time on the task (B) without earning more points (Fig. 2B). There was no group difference in reaction times (C). * $p<.05$.

Neuroscience in Psychiatry Network (NSPN) Consortium author list

Principal investigators:
Edward T. Bullmore (chief investigator from 01/01/2017)
Ian Goodyer (chief investigator until 01/01/2017)
Raymond J. Dolan
Peter Fonagy
Peter Jones

Associated faculty:
Pasco Fearon

Project managers:
Gita Prabhu

Postdoctoral research associates and associated research fellows:
Tobias U. Hauser
Michael Moutoussis
Michelle St Clair

Research assistants:
Kalia Cleridou
Hina Dadabhoy
Sian Granville
Elizabeth Harding
Alexandra Hopkins
Daniel Isaacs
Janchai King
Danae Kokorikou
Harriet Mills
Sara Pantaleone

Conflict of interest for NSPN members:
E.T.B. is employed half-time by the University of Cambridge and half-time by GlaxoSmithKline and holds stock in GlaxoSmithKline.

References:

Akaike, H., 1973. Information theory and an extension of the maximum likelihood principle, in: Petrov, B.N., Csaki, F. (Eds.), Second International Symposium on Information Theory. Budapest, pp. 267–281.

Balci, F., Simen, P., Niyogi, R., Saxe, A., Hughes, J.A., Holmes, P., Cohen, J.D., 2011. Acquisition of decision making criteria: reward rate ultimately beats accuracy. Atten. Percept. Psychophys. 73, 640–657. doi:10.3758/s13414-010-0049-7

Bishop, C., 2007. Pattern Recognition and Machine Learning. Springer, New York.

Bogacz, R., Brown, E., Moehlis, J., Holmes, P., Cohen, J.D., 2006. The physics of optimal decision making: a formal analysis of models of performance in two-alternative forced-choice tasks. Psychol. Rev. 113, 700–765. doi:10.1037/0033-295X.113.4.700

Bogacz, R., Hu, P.T., Holmes, P.J., Cohen, J.D., 2010. Do humans produce the speed-accuracy trade-off that maximizes reward rate? Q. J. Exp. Psychol. 2006 63, 863–891. doi:10.1080/17470210903091643

Burns, G.L., Keortge, S.G., Formea, G.M., Sternberger, L.G., 1996. Revision of the Padua Inventory of obsessive compulsive disorder symptoms: Distinctions between worry, obsessions, and compulsions. Behav. Res. Ther. 34, 163–173. doi:10.1016/0005-7967(95)00035-6

Cisek, P., Puskas, G.A., El-Murr, S., 2009. Decisions in changing conditions: the urgency-gating model. J. Neurosci. Off. J. Soc. Neurosci. 29, 11560–11571. doi:10.1523/JNEUROSCI.1844-09.2009

Drugowitsch, J., Moreno-Bote, R., Churchland, A.K., Shadlen, M.N., Pouget, A., 2012. The cost of accumulating evidence in perceptual decision making. J. Neurosci. Off. J. Soc. Neurosci. 32, 3612–3628. doi:10.1523/JNEUROSCI.4010-11.2012

Goldberg, D.E., 1989. Genetic Algorithms in Search, Optimization, and Machine Learning, 1 edition. ed. Addison-Wesley Professional, Reading, Mass.

Guitart-Masip, M., Huys, Q.J.M., Fuentemilla, L., Dayan, P., Duzel, E., Dolan, R.J., 2012. Go and no-go learning in reward and punishment: interactions between affect and effect. NeuroImage 62, 154–166. doi:10.1016/j.neuroimage.2012.04.024

Hauser, T.U., Moutoussis, M., Iannaccone, R., Brem, S., Walitza, S., Drechsler, R., Dayan, P., Dolan, R.J., 2017. Increased decision thresholds enhance information gathering performance in juvenile Obsessive-Compulsive Disorder (OCD). PLOS Comput. Biol. 13, e1005440. doi:10.1371/journal.pcbi.1005440

Schwarz, G., 1978. Estimating the Dimension of a Model. Ann. Stat. 6, 461–464. doi:10.1214/aos/1176344136
